# Supplementary material for: Synthesis and Characterization of N and Fe-Doped TiO2 Nanoparticles for 2,4-Dimethylaniline Mineralization
Source: Nanomaterials (Basel). 2022 Jul 24;12(15):2538. doi: 10.3390/nano12152538 (PMC9331849; doi:10.3390/nano12152538)
Supplement: Supplementary file 1 [file nanomaterials-12-02538-s001.zip › nanomaterials-1823513-supplementary.pdf]

Supplementary Materials

Synthesis and Characterization of N and Fe-Doped TiO_2 Nanoparticles for 2,4-Dimethylaniline Mineralization

Emerson Faustino [1,2,†], Thalita Ferreira da Silva [1,†], Rebeca Fabbro Cunha [1], Diego Roberto Vieira Guelfi [1], Priscila Sabioni Cavalheri [1,3], Silvio César de Oliveira [1], Anderson Rodrigues Lima Caires [4], Gleison Antonio Casagrande [1], Rodrigo Pereira Cavalcante, [1,5,*] and Amilcar Machulek Junior [1,*]

[1] Institute of Chemistry, Federal University of Mato Grosso do Sul (UFMS), Av. Senador Filinto Muller, 1555, CP 549, Campo Grande 79074-460, MS, Brazil; emerson.faustino@ifro.edu.br (E.F.); thalita.quim@gmail.com (T.F.d.S.); rebecafabbro@hotmail.com (R.F.C.); diegoguelfi@outlook.com (D.R.V.G.); priscilasabioni@hotmail.com (P.S.C.); scolive@gmail.com (S.C.d.O.); gleisoncasag@gmail.com (G.A.C.)

[2] Federal Institute of Education, Science and Technology of Rondônia, Rodovia RO-257, s/n—Zona Rural, Ariquemes, 76870-000, RO, Brazil

[3] Department of Sanitary and Environmental Engineering, Dom Bosco Catholic University, Campo Grande 79117-900, MS, Brazil

[4] Optics and Photonics Group, Institute of Physics, Federal University of Mato Grosso do Sul (UFMS), Campo Grande 79070-900, MS, Brazil; anderson.caires@ufms.br

[5] School of Technology, University of Campinas—UNICAMP, Paschoal Marmo, 1888, Limeira 13484-332, SP, Brazil

* Correspondence: rodrigoquimica14203@yahoo.com.br (R.P.C.); machulekjr@gmail.com (A.M.J.)

† These authors contributed equally to this work.

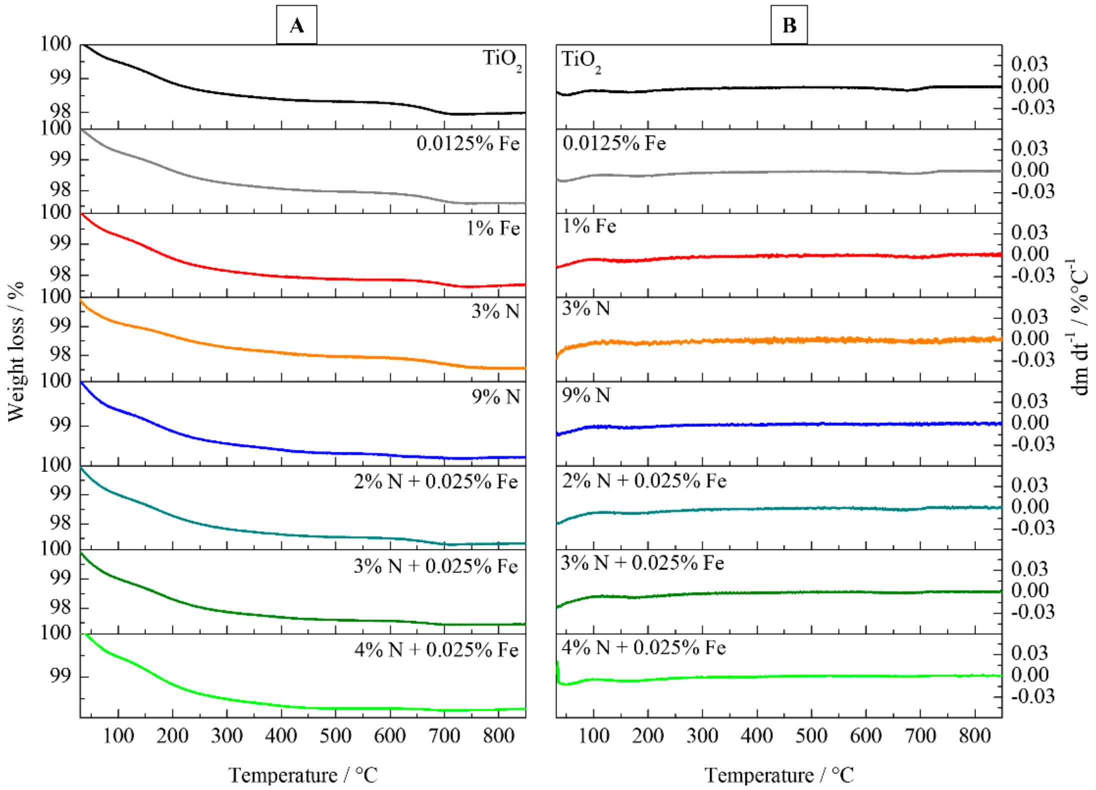

Figure S1. Thermal analysis. A) TG, and B) DTG.

Table S1. Rietveld refinement parameters for photocatalysts.

Parameters	TiO_2	0.0125% Fe	3% N
Phase	Anatase	Anatase	Anatase
a	3.783 (Å)	3.780(Å)	3.782 (Å)
b			
c	9.503 (Å)	9.496 (Å)	9.495 (Å)
α			
β	90.0000	90.0000	90.0000
γ			
Space group	I 41/amd (141)	I 41/amd (141)	I 41/amd (141)
Density	3.874 / g cm^{-3}	3.893 g cm^{-3}	3.894 g cm^{-3}
Crystal System	Tetragonal	Tetragonal	Tetragonal

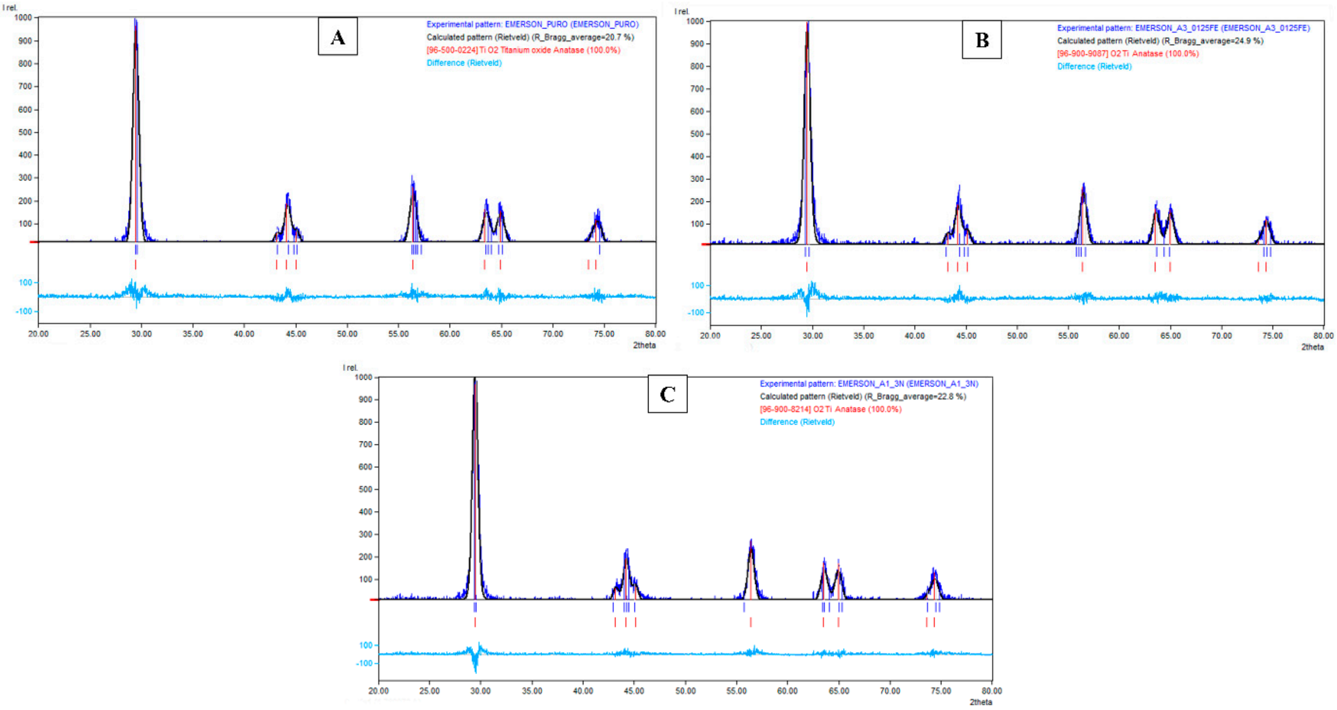

Figure S2. XRD with Rietveld refinement for TiO_2 (A), 0.125% Fe (B) and 3% N (C).

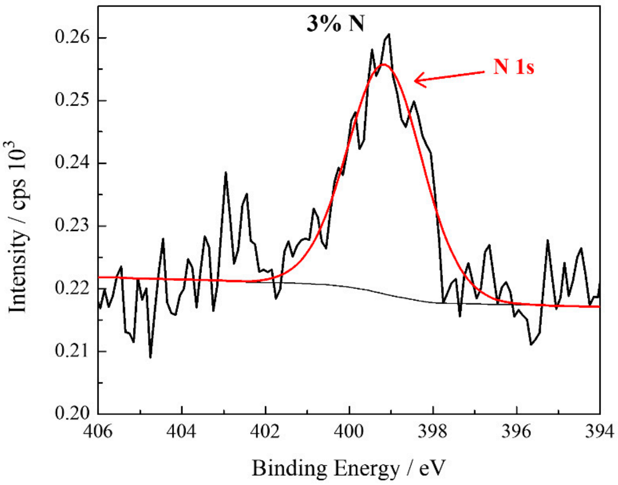

Figure S3. High-resolution XPS spectra of N 1s.

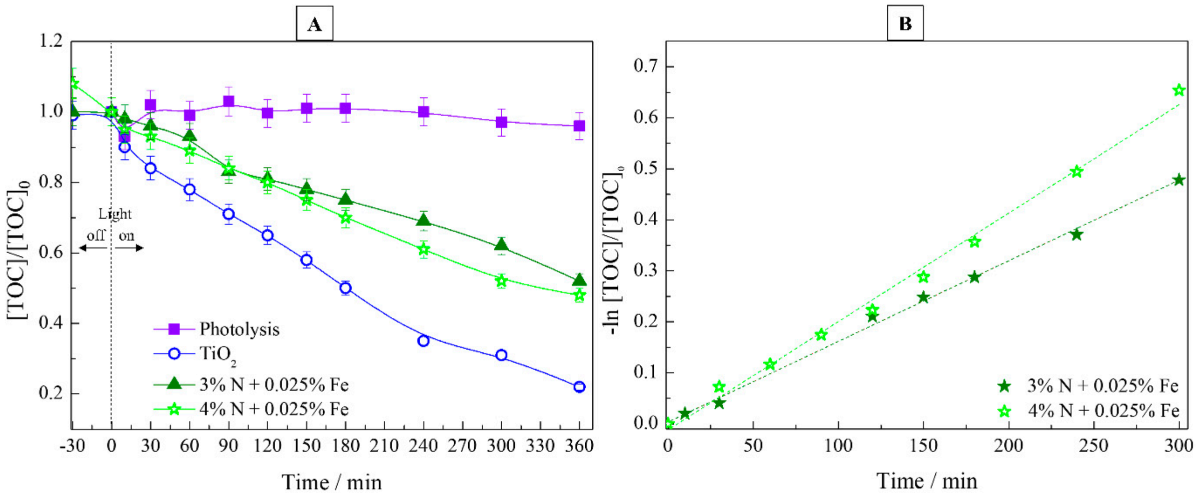

Figure S4. Results for 2,4-DMA mineralization using co-doped photocatalysts fixing 0.025%Fe and varying N doping by 3 and 4%.
